# Microstructure and Mechanical Properties of Magnesium Matrix Composites Reinforced by In Situ Reduced Graphene Oxide

**DOI:** 10.3390/ma16062303

**Published:** 2023-03-13

**Authors:** Feixiang Liu, Zhaohui Wang, Xian Du, Shubo Li, Wenbo Du

**Affiliations:** College of Materials Science and Engineering, Beijing University of Technology, Beijing 100124, China

**Keywords:** in situ reduced graphene oxide, magnesium matrix composites, microstructure, mechanical property, strengthening mechanism

## Abstract

Due to their excellent mechanical properties and large specific surface area, graphene and its derivatives are widely used in metal matrix composites as reinforcements. In this study, the thermal reduction behavior of large-size graphene oxide are investigated systematically, and reduced graphene oxide (RGO) with few residual oxygen groups and good structural integrity is obtained. ZK61 matrix composites with varying content of in situ RGO are fabricated using the semi-powder metallurgy method. The results reveal that the addition of RGO can cause the refinement of the grains and the second phase, which is attributed to the uniform distribution of the RGO throughout the matrix. The formation of nano-MgO particles is beneficial in increasing the interfacial bonding strength between the RGO and the matrix, resulting in simultaneous increments in yield strength and elongation in the RGO/ZK61 composites. The composite containing 0.6 wt.% RGO shows a superior mechanical property, including microhardness of 79.9 HV, yield strength of 203 MPa and excellent elongation of 17.5%, with increases of 20.9%, 8.6% and 7.4%, respectively, when compared with the ZK61 alloy. Quantitative analysis indicates that the main strengthening mechanisms of RGO-reinforced magnesium matrix composites are load transfer strengthening and grain refinement strengthening.

## 1. Introduction

Magnesium (Mg) and its alloys have positive application prospects in various industrial applications such as in the automobile, aerospace and electronics fields, owing to their low density, high specific strength, excellent damping capacity and electromagnetic shielding effectiveness [1,2,3]. However, their relatively low strength and poor ductility severely limit their widespread application. To overcome these disadvantages, various studies have been carried out, and the universal means of improving their mechanical properties is the addition of reinforcements such as Al_2_O_3_, SiC, AlN and carbon nanotubes (CNTs) [4,5,6,7,8]. Liang et al. reported that the fabrication of novel CNT-reinforced AZ91D composites by combining friction stir processing and ultrasonic assisted extrusion significantly enhanced the strength of Mg nanocomposites, which benefited from the good dispersion of CNTs in the matrix [8].

Compared with these nano-sized reinforcements, graphene, as a novel two-dimensional nanostructured carbon material, has attracted considerable attention owing to its excellent mechanical properties, including excellent tensile strength (130 GPa) [9], extraordinary Young’s modulus (1.02 TPa) [10] and large surface area (2630 m^2^/g) [11]. Therefore, graphene has been considered as a promising reinforcement to improve the mechanical properties of Mg matrix composites. Many investigations have been carried out to study the microstructure evolution, properties, as well as the preparation process of graphene-reinforced Mg-based composites [12,13,14,15]. The properties of these composites were found to be much lower than the expected values. On the one hand, achieving the uniform dispersion of graphene in Mg matrix is a great challenge due to the strong van der Waals forces between the layers, caused by the large specific surface area. On the other hand, the higher tendency to agglomeration, except at elevated temperature, and the lower wettability damage the continuity of the Mg matrix and reduces the interfacial bonding between the graphene and the matrix. Both of these degrade the strengthening effects of the graphene on the mechanical properties of the Mg matrix composites. Many techniques have been used to disperse graphene in the Mg matrix and to improve the interfacial bonding, such as ultrasonic processing [16], stir melting [17], ball milling [18], in situ reaction [19] and chemical coating [20]. However, some of these methods may damage the structure and the integrity of the graphene (e.g., high-temperature stirring and high-energy ball milling) and degrade their strengthening effect on Mg matrix composites.

Graphene oxide (GO) is an oxidized derivative of graphene that contains hydroxyl (-OH), carboxyl (-COOH), carbonyl (C=O) and epoxy (C-O-C) groups on the surface. The presence of these oxygen-containing functional groups makes GO much easier to disperse in solvents (deionized water, ethanol, etc.) and to distribute uniformly in the composites. Moreover, GO is easier to absorb onto metallic substrates by electrostatic adsorption, thereby significantly improving the interfacial bonding with the matrix. These advantages suggest it has potential to improve the mechanical properties of Mg alloys by the addition of GO, and several studies investigating this have been carried out [21,22]. Nevertheless, the oxygen in the GO has a negative impact on the interfacial structure and mechanical properties of the GO-reinforced Mg matrix composites due to the formation of excessive brittle MgO between the GO and the Mg matrix. Therefore, it is necessary to develop a novel process to improve the dispersion of the GO and decrease the oxidation to achieve the desired enhancement effects.

In this study, the thermal reduction properties of large-size GO are systematically investigated and are used to obtain reduced graphene oxide (RGO) with few residual oxygen groups and without damage to the graphene structure. ZK61 alloy powders mixed with different amounts of in situ RGO are fabricated by solution mixing, reduced-pressure distillation and thermal reduction. Then in situ RGO-reinforced ZK61 matrix composites are successfully prepared by hot-press sintering and hot extruding. The influence of RGO on the microstructure characteristics and mechanical properties of the ZK61 alloy is investigated in detail and the strengthening mechanisms are discussed with quantitative analysis.

## 2. Experimental Procedures

### 2.1. Fabrication of Composites

ZK61 magnesium alloy powder with a particle diameter of 50–80 μm was provided by Nantong Jinyuan Intelligent Technology Co., Ltd., Jiangsu, China. The chemical composition of the ZK61 magnesium alloy was Mg-5.2Zn-0.3Zr (wt.%). GO with a thickness of 1.2–4.0 nm, diameter of 30–70 μm and purity of >98% was obtained from the Beijing Institute of Aeronautical Materials, China.

**Figure 1:**
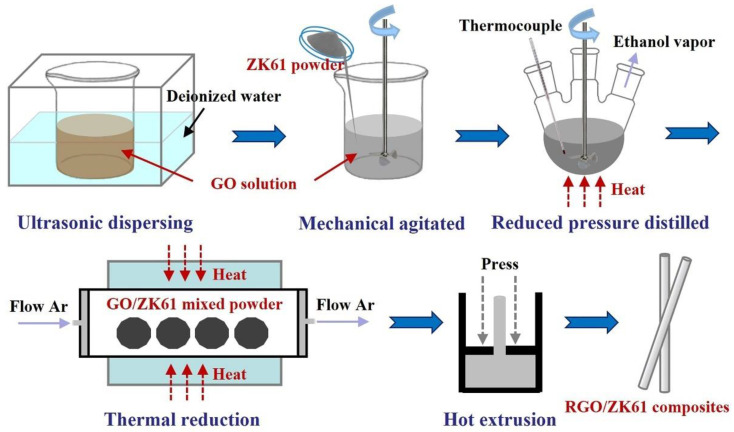
Schematic diagram of the fabrication of RGO/ZK61 composites.

The RGO-reinforced magnesium matrix composites were fabricated using the semi-powder metallurgy method, as shown in Figure 1. First, the GO was separately ultrasonicated in ethanol with frequency of 40 kHz for 90 min to obtain a stable suspension. Then, the ZK61 powder was slowly put into the suspension to form a uniform mixture, which was mechanically agitated continuously at 300–500 rpm for 60 min. After that, the ethanol in the mixture was removed by reduced pressure distillation at 343 K and then thermally reduced at 773 K for 2 h to obtain RGO/ZK61 composite powders with in situ RGO amounts of 0.1 wt.%, 0.25 wt.%, 0.4 wt.% and 0.6 wt.%. The mixed powders were sintered by the hot-press sintering method in a stainless steel mold at 473 K under 200 MPa pressure to obtain green billets. Finally, the composite powders were hot extruded at 623 K at an extrusion speed of 1 mm/s and an extrusion ratio of 25:1 to obtain the RGO-reinforced magnesium matrix composites. The size of the extruded composites was Φ8 mm × 180 mm, where the length parallel to the ED direction was 180 mm. For comparison, ZK61 alloy without RGO was fabricated using the identity procedure.

### 2.2. Characterization

#### 2.2.1. GO and RGO

To characterize the thermal reduction of the GO sample, differential scanning calorimetry (DSC) and thermogravimetric analysis (TGA) were measured in a NETZSCH-449C Simultaneous Thermal Analyzer, which were performed from room temperature to 873 K with a heating rate of 5 K/min under a flowing argon atmosphere. The Raman spectroscopy was recorded from 1000 cm^−1^ to 3500 cm^−1^ on a JY HR800 with a 532 nm argon ion laser (0.1 mW). The oxygen-containing group information was measured using a Nicolet Nexus 670 type Fourier transform infrared spectroscopy (FTIR) instrument. Morphology features were characterized by using scanning electron microscopy (SEM, HITACHI SU-8020) and high-resolution transmission electron microscopy (HRTEM, JEOL JEM-2100).

#### 2.2.2. ZK61 Alloy and RGO/ZK61 Composites

The ZK61 alloy and RGO/ZK61 composites were mechanically sanded with a series of SiC (600#, 1000#, 2000#, 3000# and 5000#) and polished with diamond paste for the microstructure observation. Phase analysis was performed by X-ray diffraction (XRD, D/MAX-3C) using a diffractometer equipped with Cu Kα radiation and a scan rate of 0.02°/s in 2θ range of 10–90°. Microstructural characterization was investigated using a Zeiss Axio Imager A2m type optical microscope (OM), a HITACHI SU-8020 SEM equipped with energy dispersive spectrometer (EDS) and a JEOL JEM-2100 HRTEM. Thin foil specimens for HRTEM observation were prepared by mechanical polishing, disc punching and ion thinning using a Gatan precision ion polishing system (GATAN691).

A Vickers microhardness test was conducted using an HVS-1000 hardness tester under a load of 100 g held for 10 s. At least six indentations of microhardness were taken for each sample and the average was calculated to minimize experimental error. Tensile testing was carried out using a DNS-20 universal testing machine under a constant speed of 1 mm/min. Samples for the tensile test were made into dog bones with a size of 4 mm gauge diameter and 25 mm gauge length [23], which were sectioned in parallel to the extrusion direction. The fractured surfaces were observed on a HITACHI SU-8020 SEM.

## 3. Results and Discussion

### 3.1. Characterization of Raw Materials and Composite Powders

**Figure 2:**
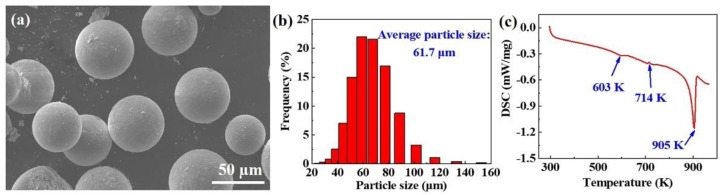
(**a**) SEM image of ZK61 powder and corresponding (**b**) particle size distribution with an average size of 61.7 μm; and (**c**) DSC curve of the ZK61 sample.

Figure 2a,b demonstrate the SEM morphology and particle size distribution of the ZK61 powder. It can be seen that the ZK61 powder shows a regular spherical morphology with an average particle size of 61.7 μm. The surface is clean and has no obvious oxide or impurities. The DSC curve of the ZK61 powder during the melting process (Figure 2c) shows two endothermic peaks at about 603 K and 905 K, which are related to the melting of the MgZn phase [24] and the α-Mg, respectively. In addition, there is an exothermic peak at about 714 K, which is associated with the transformation of the second phase. It can also be seen from Figure 2c that the melting of the α-Mg starts at approximately 800 K. This result provides a reference for the temperature of the thermal reduction of GO and the hot extrusion, which should be below 800 K.

**Figure 3:**
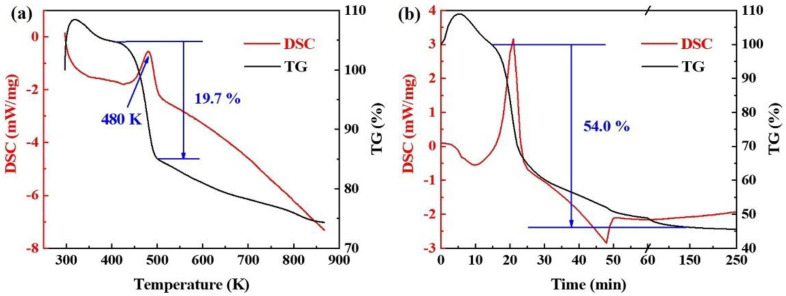
Differential scanning calorimetry of graphene oxide with (**a**) increasing temperature to 873 K and (**b**) increasing to 773 K and holding for 2 h.

DSC/TGA analysis was conducted to investigate the thermal properties of the GO, as shown in Figure 3. The DSC curve in Figure 3a shows that a wide exothermic peak exists at around 480 K, which corresponds to the thermal decomposition of the unstable oxygen-containing functional groups. In this stage, about 19.7% mass loss occurred owing to the degassing of the CO, CO_2_ and H_2_O produced by the pyrolysis of the oxygen-containing functional groups such as -OH, -COOH and -C=O [25,26]. The mass loss of GO shows a stable trend with the increase of temperature, that is, about 2.5% mass loss per 100 K, indicating the final decomposition of the oxygen-containing functional groups. Considering the initial temperature of the melting of the α-Mg, we heated the GO to 773 K and held it for 2 h (shown in Figure 3b) to further analyze the effect of the reduction time on the thermal decomposition behavior of the GO. The thermal decomposition in the heating stage is consistent with Figure 3a, and the effect of time on the thermal properties of the GO is insignificant when compared with that of the temperature. No obvious variation in mass loss was seen during the holding stage, and the value eventually reached about 54.0%. These results show that GO might be significantly reduced at 773 K for 2 h.

**Figure 4:**
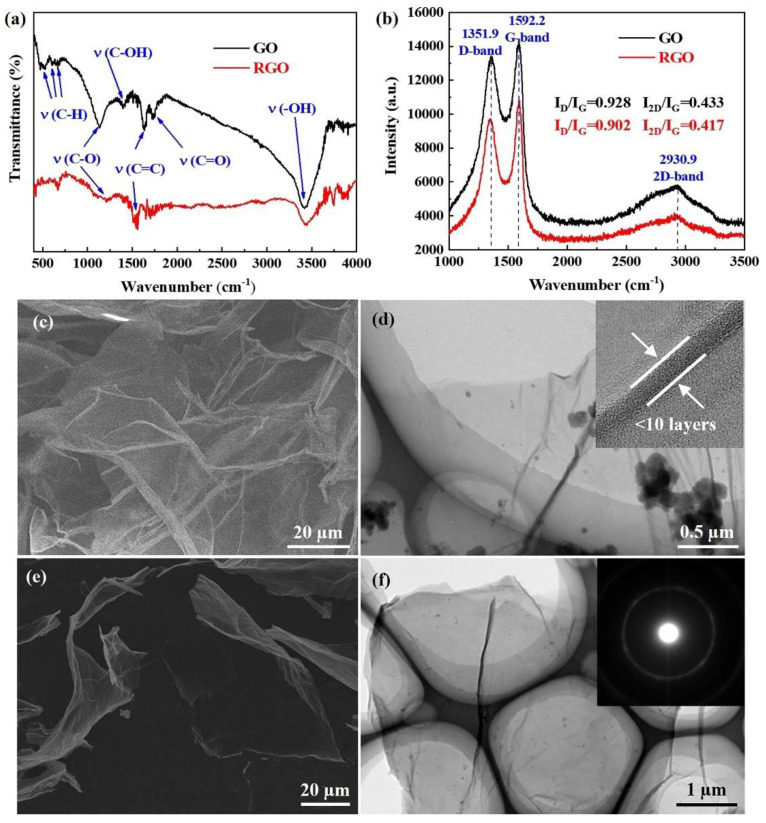
(**a**) FTIR spectrum and (**b**) Raman spectroscopy of GO and RGO. SEM and TEM micrographs of (**c**,**d**) GO and (**e**,**f**) RGO.

FTIR analysis is regarded as an effective method to determine the functional groups of GO and RGO, and then to show the reduction degree of GO. Figure 4a shows the FTIR spectroscopy analysis of the GO and RGO, which was thermally reduced at 773 K for 2 h. The spectrum reveals that the GO consists of a variety of oxygen-containing functional groups that includes C-H, C-O, C-OH, C=O and -OH groups [27,28,29]. It can be seen that the peaks from 460–670 cm^−1^ correspond to the bending vibration bands of the C-H groups. A sharp peak that exists at 1132 cm^−1^ represents the C-O stretching of the epoxy groups, and the peaks at 1398 cm^−1^ and 1727 cm^−1^ reveal the vibration absorption peak of the C-OH and the stretching vibration band of the C=O, respectively. The broad peak near 3357 cm^−1^ is attributed to the stretching and bending vibration of the -OH groups due to the presence of water molecules. In addition, the spectra show a stretching vibration C=C peak at 1623 cm^−1^ corresponding to the remaining sp^2^ character. Comparing the FTIR spectra of the GO, the characteristic peaks of the oxygen-containing functional groups in the RGO show varying degrees of reduction after thermal reduction. The peaks of C-OH and C=O tends to disappear and the intensity of the C-O and -OH decreased significantly. The results show that the thermal reduction process could remove most of the oxygen-containing functional groups in GO.

The Raman spectrum contains three prominent peaks at around 1351.9 cm^−1^, 1592.2 cm^−1^ and 2930.9 cm^−1^, which correspond to the D, G and 2D bands, respectively, as shown in Figure 4b. There is a weak 2D band and a strong D band with an intensity comparable to that of the G band in both the GO and RGO. The D band is associated with the breathing mode of six-atom aromatic rings arising due to defects and disorders in the sample. The intensity of the D band is therefore used to measure the degree of disorders [30]. The G band correlation with the optical E2g phonons at the Brillouin zone center is caused by the bond stretching of all the pairs of sp^2^ carbon pairs in both the rings and chains. The 2D band, also called the G band, is related to the set of graphene layers [29]. It can be seen from Figure 4b that the intensity of the D band relative to the G band (*I_D_*/*I_G_*) decreases slightly from 0.928 to 0.902, reflecting the abundance of defects in the GO and RGO. In addition, the ratio of the intensities of the 2D band and G band (*I*_2*D*_/*I_G_*) of the GO and RGO is 0.433 and 0.417, respectively, indicating that the RGO retains the same multi-layer structure as the GO. Therefore, the Raman analysis provides further evidence for the significant reduction of GO in these conditions.

The microstructures of the GO and RGO after ultrasonic dispersing were analyzed using SEM and TEM, as represented in Figure 4c–f. The intercalation of the oxygen-containing functional groups on the GO could destroy the π-π conjugated structure, resulting in poor electrical conductivity. Therefore, the morphology of the GO under SEM is blurred (shown in Figure 3a). The individual GO sheet is found to have a width of 30–70 μm, which is much larger than that of other metal matrix composites [31,32,33]. From Figure 4d we can see that the GO is transparent and slightly aggregated, with the wrinkles loosely distributed on the surface. It can also be noted that the GO sheet is thinner (fewer than 10 layers), with a monolayer thickness of 0.34 nm and a layer spacing of 0.416 nm. After reduction at 773 K for 2 h, the oxygen-containing functional groups on the RGO have been removed and the π-π conjugated structure has recovered significantly, resulting in clear morphology under SEM observation (Figure 4e). In addition, the RGO exhibits a smooth surface and the strong van der Waals force between the layers increases the agglomeration and the monolayer thickness, as shown in Figure 4f. The corresponding selected area electron diffraction (SAED) result reveals the polycrystal feature of the RGO.

**Figure 5:**
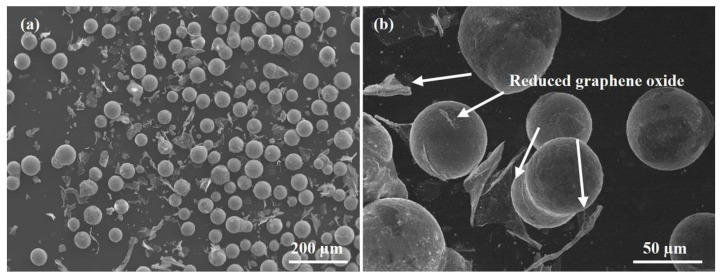
(**a**) SEM image of RGO/ZK61 composite powder and (**b**) details of RGO dispersed in magnesium matrix composite powder.

The SEM images of the ZK61 powder with the addition of the RGO are shown in Figure 5. It can be seen that the RGO sheets are dispersed uniformly in the mixed powder and no obvious clusters are observed. Parts of the RGO are distributed among the alloy powders and the others are well spread on the surface of the particles. This indicates that semi-powder metallurgy technology is highly effective in uniformly dispersing RGO in the magnesium matrix. On the one hand, the residual oxygen-containing functional groups on the RGO are assisted to disperse homogeneously through the mechanical stirring and the ultrasonic process. On the other hand, the friction resistance and electrostatic adsorption between the RGO and ZK61 powders are helpful to adhere the RGO to the surface of the ZK61 particles.

### 3.2. Microstructures

**Figure 6:**
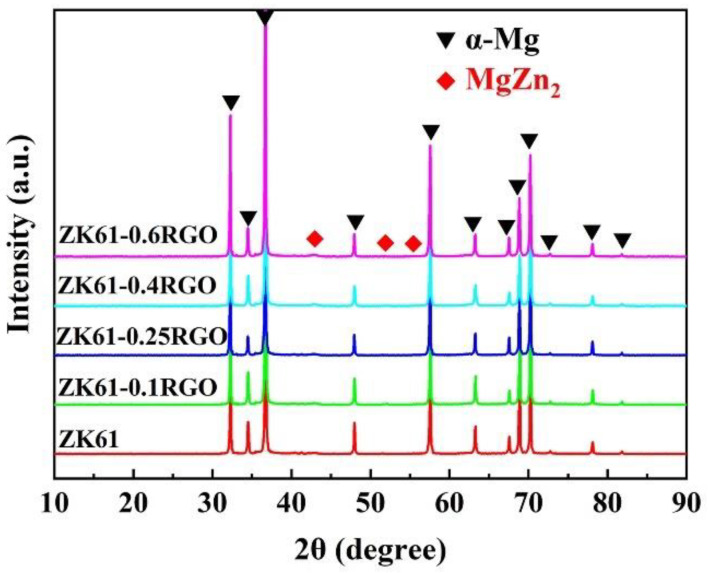
X-ray diffraction patterns of RGO-reinforced ZK61 matrix composites with different addition of RGO.

Figure 6 shows the XRD patterns of the RGO-reinforced ZK61 matrix composites with different additions of RGO. The α-Mg phase and β-MgZn_2_ phase are detected in both the ZK61 alloy and its composites, indicating that the phase transformation occurred during the preparation process. This phenomenon is closely in accordance with the DSC/TGA results of the ZK61 powder. In addition, no characteristic peak of the new phase is detected, and the diffraction peaks of α-Mg and β-MgZn_2_ have no broadened or shifted with the addition of the RGO. No characteristic peak of the RGO at 2θ = 23.8° [34] is detected even in the composite added with 0.6 wt.% RGO, which is probably due to the lower amount and the good dispersion of the RGO in the RGO/ZK61 composites, meaning that the XRD cannot identify it.

**Figure 7:**
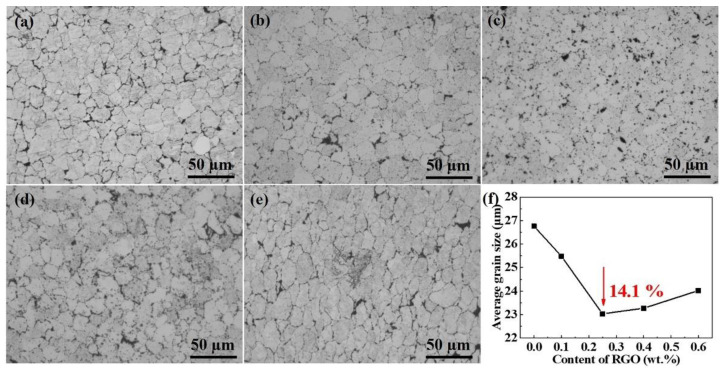
Microstructure of (**a**) ZK61 alloy and its composites containing (**b**) 0.1 wt.%, (**c**) 0.25 wt.%, (**d**) 0.4 wt.% and (**e**) 0.6 wt.% RGO. (**f**) The average grain size of RGO-reinforced magnesium matrix composites with different RGO content.

To verify the effects of the RGO on the microstructure evolution of the RGO/ZK61 composites, the optical images of the samples were analyzed, as shown in Figure 7. As can be seen from Figure 7a, the grains in the ZK61 matrix are equiaxed and some dark spots are uniformly dispersed on the grain boundaries, which could be related to the preferred precipitation of the MgZn_2_ phase. The addition of the RGO refined the grains of the ZK61 alloy, and the average grain size decreased gradually as the content of RGO was increased, reaching 23.0 µm by 14.2% with 0.25 wt.% RGO. It can be seen that the RGO was highly effective in refining the grains of the ZK61 alloy. RGO with a 2D lamellar structure can act as a nucleation substrate and enhance heterogeneous nucleation, which effectively impedes the atomic diffusion and grain growth and obtains a finer microstructure [21]. However, the mean grain size increases slightly with further increases in the amount of RGO, which is mainly owing to the poor grain refinement caused by the RGO clusters on these composites.

**Figure 8:**
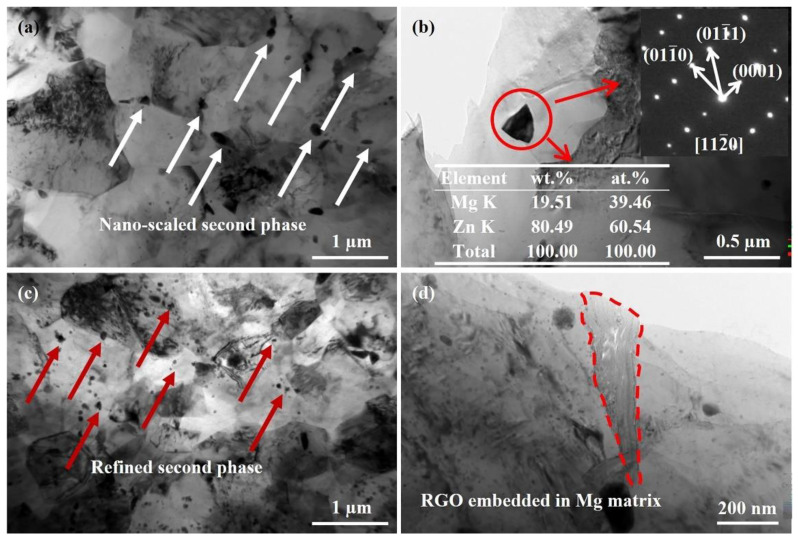
TEM micrographs of (**a**,**b**) ZK61 alloy and (**c**,**d**) 0.6 wt.% RGO/ZK61 composite.

The morphology of RGO and the interfacial relationship between RGO and the matrix are difficult to characterize using OM and SEM due to the 2D lamellar structure of RGO with nanoscale thickness. Therefore, TEM images of the ZK61 alloy and its composites were analyzed, and are shown in Figure 8. The grain size of the matrix alloy is small, and the second phase is uniformly distributed in the grain interiors and on the grain boundaries, with the size in nanoscale (as indicated by the arrow in Figure 8a). The EDS result of the second phase reveals that the atomic ratio of Mg and Zn is about 1:2. The corresponding SAED pattern indicates that the precipitate has a hexagonal close-packed structure and can be identified as MgZn_2_ phase. Figure 8c shows that the addition of the RGO has an obvious refining effect on the grain and the second phase, which has the benefit of improving the mechanical properties of the ZK61 alloy. In addition, the integrity of RGO sheet can be observed in the RGO/ZK61 composite (Figure 8d), indicating the graphene structure remained intact. The interface between the RGO and magnesium is well bonded, and no porosity, voids, cracks and other defects are observed.

**Figure 9:**
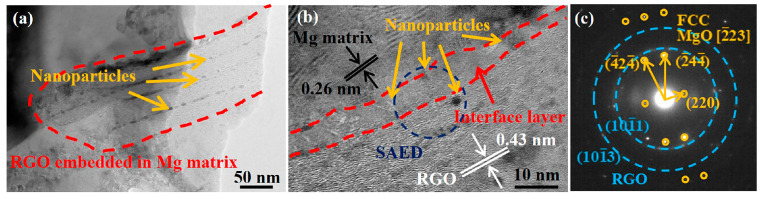
(**a**) RGO embedded in 0.6 wt.% RGO/ZK61 composite with nanoparticles distributed on the surface of RGO; (**b**) interfacial structure between RGO and magnesium matrix; and (**c**) selected area electron diffraction of the interface layer in (**b**).

To further clarify the interaction mechanism of the RGO and the magnesium matrix, the interfacial structure between the RGO and the matrix was analyzed, as shown in Figure 9. Figure 9a presents the TEM observation of typical RGO embedded in the Mg matrix, revealing a thin, wrinkled, paper-like structure. An interfacial layer between the RGO and magnesium matrix is observed. In addition, there are a few nanoscale particles distributed on the surface of the RGO. In order to reveal more details of the nanoparticles and the interfacial structure between the magnesium matrix and the RGO, an HRTEM image is provided in Figure 9b. It can be clearly seen that the thickness of the interfacial layer is about 5 nm and the nanoparticles are mainly distributed around the interface. The lattice space of the left side of the interfacial layer is about 0.26 nm, which corresponds to the (0002) plane of the Mg matrix. The lattice fringes of the right side of the interfacial layer exhibit an interplanar spacing of 0.43 nm, which is consistent with that of the initial RGO. This indicates that the RGO maintained structural integrity during the fabrication process, which would help to enhance the mechanical interlocking and load transfer between the RGO and Mg matrix. The SAED pattern of the selected region in Figure 9b shows that these nanoparticles are MgO (shown in Figure 9c), suggesting that the interfacial reaction occurred between the magnesium and the residual oxygen in the RGO. The MgO nanoparticles are formed during the mixing process because of the reaction of the RGO and the magnesium matrix, and are mainly distributed along the extrusion direction due to the extrusion deformation (as shown in Figure 9a). The existence of the MgO nanoparticles at the interface plays a transitional function, which is helpful in improving the interfacial bonding between the RGO and the magnesium matrix.

### 3.3. Mechanical Properties

**Figure 10:**
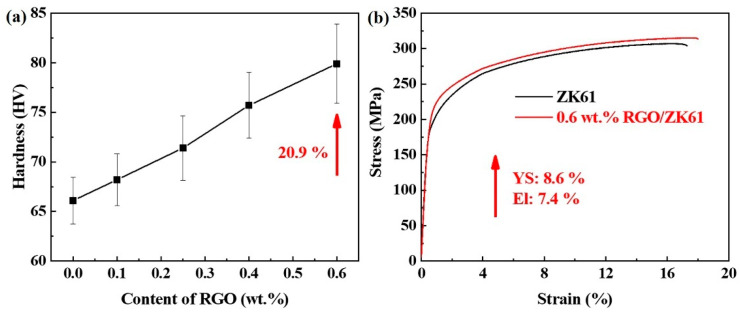
(**a**) Vickers hardness of RGO-reinforced ZK61 matrix composites with increasing content of RGO. (**b**) Tensile stress–strain curves of ZK61 alloy and the composite with 0.6 wt.% RGO addition.

Figure 10 shows the microhardness and representative engineering tensile stress–strain curves of the ZK61 alloy and its composites. The values of yield strength (*YS*), ultimate tensile strength (*UTS*), elongation (*ε*) and Vicker hardness (*HV*) are listed in Table 1. The hardness of the RGO/ZK61 composites increases gradually with increases in the content of RGO, and achieves a peak value of 79.9 HV at 0.6 wt.%, representing a 20.9% improvement on that of the ZK61 alloy. Meanwhile, the yield strength is enhanced progressively in the same way as the hardness, with a value of 203 MPa, which is 8.6% higher than the matrix alloy for the composite added with 0.6 wt.% RGO. A finer grain size and grain refinement are effective in improving the mechanical properties of the RGO-reinforced ZK61 matrix composites. In addition, the uniform distribution of the RGO in the matrix effectively improves the deformation resistance of the composites, resulting in a significant increment in the microhardness and yield strength. More importantly, the interfacial bonding between the RGO and α-Mg is significantly strengthened by the MgO nanoparticles, which evidently improved the enhancement effect of the RGO. It can be seen from Table 1 that the tensile strength and elongation decreased gradually with the addition of 0.1 wt.% and 0.25 wt.% RGO. As the addition of RGO is lower, the efficiency of the load transfer is poor, and dislocation will be accumulated around the RGO, leading to the earlier fracture of the sample. With increasing RGO content, the network structure seems to be easily formed and obtains a strong interfacial bonding between the RGO and Mg matrix. This makes the stress transfer effectively from the soft Mg to the hard RGO, resulting in a significant enhancement in both the strength and elongation of these composites.

### 3.4. Strengthening Mechanisms

As mentioned above, the RGO-reinforced magnesium matrix composites show a superior mechanical property when compared with the ZK61 alloy, which is mainly caused by the following factors: grain refinement strengthening, thermal mismatch strengthening, Orowan strengthening and load transfer strengthening. As presented in Figure 7, the RGO can refine the grains of the ZK61 alloy effectively, which is beneficial to improving yield strength. The relationship between yield strength and grain size consistent with the Hall–Petch equation [35,36], and the contribution of grain refinement to strength, Δ*σ_H−P_*, can be explained by:

(1) $$∆\sigma_{H-P}=K\left(d_{c}^{-\frac{1}{2}}-d_{m}^{-\frac{1}{2}}\right),$$

where *d_m_* and *d_c_* are the average grain size of the ZK61 alloy and its composites, and *K* is the Hall–Petch coefficient (130 MPa·μm^1/2^ for magnesium alloy [17]).

Due to the difference in the coefficients of thermal expansion (CTE) and elastic modulus between the RGO and magnesium matrix [37], the hardening occurs and a high density of dislocations form during the preparation process. The hard RGO will restrict the movement of the dislocations, thus improving the strength of the composites. The strengthening of the composites due to the mismatch in CTE can be expressed as [38]:

(2) $$∆\sigma_{CTE}=\beta \mu_{m}b\sqrt{\frac{8f∆\alpha ∆T}{\left(1-f\right)b\bar{d}}},$$

where *β* is a geometric constant (1.25), *b* is Burger’s vector of the Mg matrix (0.321 nm), *f* is the volume fraction of the RGO, Δ*α* is the difference in CTE between the RGO and matrix [39], Δ*T* is the difference between the extrusion and test temperatures, $\bar{d}$ is the average diameter of RGO, and *μ_m_* is the shear modulus of the matrix and can be calculated using Equation (3):

(3) $$\mu_{m}=\frac{E}{2\left(1+\nu_{m}\right)},$$

where *ν_m_* is the Poisson’s ratio (0.35 for the Mg matrix [40]) and *E* is the elastic modulus of the matrix, which can be obtained by the tensile stress–strain curve.

Based on the shear-lag model [41], load can be efficiently transferred to graphene through shear stress at the interface, thus playing an important role in strengthening the composites. The enhancement effect is mainly related to the dispersion, volume fraction, average size and interface wettability of RGO. The increase in yield strength due to load transfer can be estimated by a modified shear-lag model [42]:

(4) $$∆\sigma_{LT}=f\left(\frac{S}{A}\right)\left(\frac{\tau_{m}}{2}\right)-f\sigma_{m},$$

where *σ_m_* is the yield strength of the matrix, *τ_m_* is the shear strength of the matrix, equal to *σ_m_*/2. *S* and *A* are the interfacial area and cross-sectional area of the RGO sheet and can be calculated by 2(*l* + *t*)*l* and *l* × *t*, respectively, where *l* and *t* are the side length and thickness of the RGO.

Owing to the addition of graphene to the Mg matrix, residual dislocation loops formed around the graphene when the dislocations pass through the graphene during the deformation. Orowan loops around the graphene can increase the deformation resistance, thereby improving the strength of the composite. Thus, research reveals that Orowan strengthening plays an important role in metal matrix composites [43]. However, the RGO used in this study is in microscale, meaning that it is difficult to form Orowan loops and inhibit the dislocation movement. Therefore, Orowan strengthening can be ignored in this work.

Thus, the increment of yield strength for the composites can be estimated as Δ*σ* = Δ*σ_H−P_* + Δ*σ_CTE_* + Δ*σ_LT_*. However, RGO is randomly distributed in composites, and the distribution state significantly affects the strengthening efficiency of the load transfer strengthening. Earlier research has reported that plated graphene arranged at large angles (from 45° to 90°) along the tensile direction would not contribute significantly to the yield strength of the composites [44]. Therefore, the improved yield strength contributed by load transfer strengthening can be calculated as:

Δ*σ_LT_* = Δ*σ* − Δ*σ_H−P_* − Δ*σ_CTE_*, (5)

**Figure 11:**
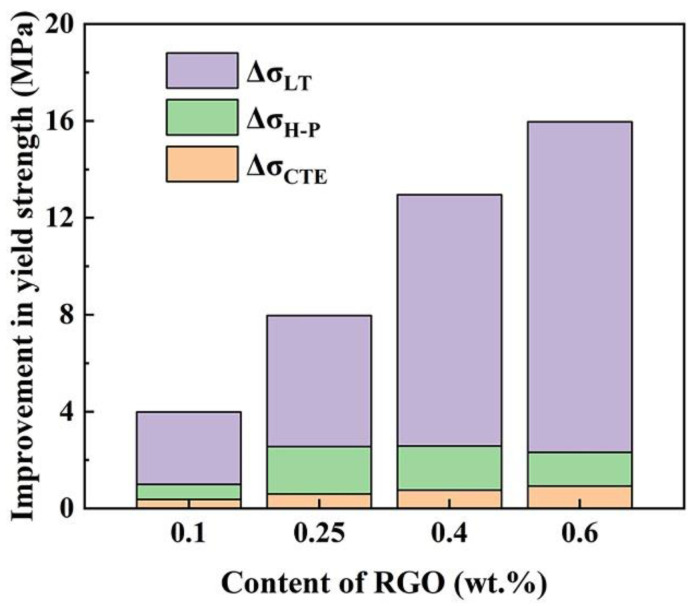
Contributions of each strengthening effect to RGO/ZK61 composites.

Table 2 and Figure 11 present the calculated contributions and proportion of each strengthening mechanism to the yield strength of the RGO/ZK61 composites based on Equations (1)–(5). The calculations reveal that the contribution of the load transfer strengthening induced by the RGO is much higher than the other strengthening factors, which means that load transfer strengthening is the main strengthening mechanism in the RGO-reinforced ZK61 magnesium matrix composites. The value increases linearly with the increase of RGO content, to occupy 68.0% to 85.5% of the total contributions. It can be seen that the grain refinement strengthening effect first increased and then decreased slightly with increasing RGO content, which is related to the refined grains in the composites, accounting for 8.7% to 24.5% of the total strengthening effect. Due to the larger diameter and lower volume fraction of RGO, the thermal mismatch strengthening effect is weak, but increases gradually with increasing RGO content.

**Figure 12:**
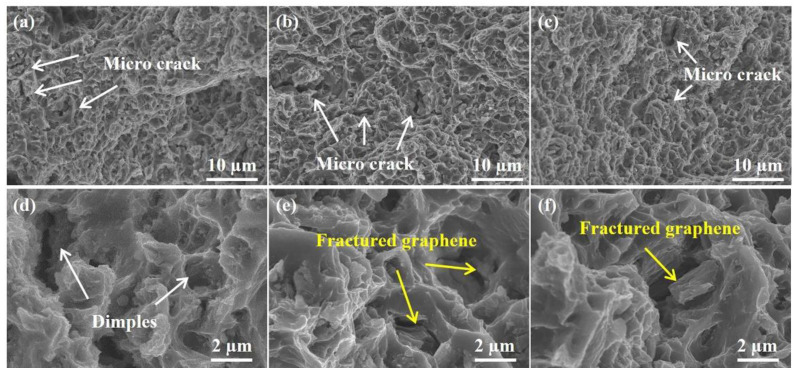
Tensile fracture morphologies of (**a**,**d**) ZK61 alloy, (**b**,**e**) 0.4 wt.% RGO/ZK61 composite and (**c**,**f**) 0.6 wt.% RGO/ZK61 composite.

The tensile fracture morphologies of the ZK61 alloy and its composites were analyzed to further reveal the strengthening effect of RGO, as shown in Figure 12. It can be seen that the ZK61 matrix alloy exhibits plenty of dimples and a few microcracks and tear ridges along the tensile direction, indicating a ductile characteristic of the fracture. In the case of the 0.4 wt.% RGO/ZK61 composite, the fracture displays larger and deeper dimples and higher tear edges. This may be related to the weaker efficiency of the load transfer and the dislocation stacking near the interface due to the lower RGO content. Despite this, the formation of nano-MgO particles could improve the interfacial bonding and then enhance the reinforcement of the RGO. Therefore, the reduction in elongation occurs in these composites while increasing yield strength and harness (as shown in Table 1). The dimple width and tearing edges decreased with increasing RGO content to 0.6 wt.%. In addition, RGO distributed along the stress direction is pulled out from the matrix during the tensile process, as shown in Figure 12f. The stress can be transferred to high-strength RGO effectively, thereby promoting strength and elongation simultaneously.

## 4. Conclusions

ZK61 matrix composites reinforced by in situ reduced graphene oxide have been successfully fabricated using the semi-powder metallurgy method. The thermal reduction properties of large-size GO have been systematically studied. The effect of RGO on the microstructure and mechanical properties of the composites have been analyzed, and the strengthening mechanisms of these composites have then been obtained. The following conclusions can be drawn:

1. Most of the oxygen-containing functional groups in GO can be removed through thermal reduction at 773 K for 2 h, with structural integrity well maintained.
2. Microstructural characterization of the composites reveals that the grains and second phase are clearly refined with the addition of RGO. The in situ nano-MgO particles are formed around the interface owing to the few residual oxygen-containing groups on the surface of the RGO. The chemical bonding between the RGO and the magnesium matrix significantly improved the interfacial bonding, and then enhanced the reinforcement of the RGO.
3. The addition of in situ RGO significantly improves the mechanical properties of the composites. The microhardness, yield strength and elongation of the 0.6 wt.% RGO/ZK61 composite are optimal among the samples at 79.9 HV, 203 MPa and 17.5%, respectively, and which increased by approximately 20.9%, 8.6% and 7.4%, respectively, compared with those of the ZK61 alloy.
4. Due to the strong interfacial bonding and the refined grains in the RGO-reinforced ZK61 magnesium matrix composites, load transfer strengthening and grain refinement are the main strengthening mechanisms, which caused the enhancement of strength and elongation simultaneously. The effects of thermal mismatch and Orowan strengthening on the mechanical properties are insignificant.

## Figures and Tables

**Table 1:** Mechanical properties of RGO/ZK61 composites at ambient temperature.

Wt.% of RGO	*YS* (MPa)	*UTS* (MPa)	*ε* (%)	Hardness (HV)
0	187 ± 1	307 ± 1	15.9 ± 0.7	66.1 ± 2.4
0.1	191 ± 2	307 ± 0	14.9 ± 0.6	68.2 ± 2.6
0.25	195 ± 1	298 ± 1	9.4 ± 0.9	71.4 ± 3.3
0.4	200 ± 2	301 ± 0	9.8 ± 0.7	75.7 ± 3.3
0.6	203 ± 2	312 ± 3	17.5 ± 0.4	79.9 ± 4.0

**Table 2:** Calculated contributions and proportion of each strengthening mechanism to the yield strength of RGO/ZK61 composites.

Wt.% of RGO	Δ*σ_CTE_* (MPa)	Δ*σ_H-P_* (MPa)	Δ*σ_LT_* (MPa)	Δ*σ* (MPa)
0.1	0.38 (9.5%)	0.62 (15.5%)	3.00 (75.0%)	4
0.25	0.6 (7.5%)	1.96 (24.5%)	5.44 (68.0%)	8
0.4	0.76 (5.8%)	1.82 (14.0%)	10.42 (80.2%)	13
0.6	0.93 (5.8%)	1.39 (8.7%)	13.68 (85.5%)	16

## Data Availability

Not applicable.
